# Distinct Routes to Understand the Relationship Between Dispositional Optimism and Life Satisfaction: Self-Control and Grit, Positive Affect, Gratitude, and Meaning in Life

**DOI:** 10.3389/fpsyg.2020.00907

**Published:** 2020-05-26

**Authors:** Xavier Oriol, Rafael Miranda, César Bazán, Estefany Benavente

**Affiliations:** ^1^Faculty of Education and Social Science, Andres Bello University, Santiago, Chile; ^2^Department of Psychology, Continental University, Huancayo, Peru; ^3^Universidad Marcelino Champagnat, Lima, Peru; ^4^Ministerio de Educación del Perú, Lima, Peru

**Keywords:** dispositional optimism, life satisfaction, self-control and grit, positive affect, gratitude, meaning in life

## Abstract

Over the last years, understanding the implications of prospective thinking toward the future has become of increasing interest. This study aims to delve into the relation between dispositional optimism, one of the most relevant prospective constructs, and life satisfaction. Additionally, we also seek to prove the mediating effect of different cognitive and affective variables associated with both hedonic and eudaimonic well-being on this relationship. A first study is conducted with 275 secondary students to assess the relationship between optimism and life satisfaction through self-control and grit (14.82, SD = 1.07), which indicates a mediating effect of grit but not of self-control. A second study is carried out with 1,356 university students (21.5, SD = 2.35) to demonstrate the mediating effect of positive affect on dispositional optimism and life satisfaction. Results show a strong relationship between optimism and positive affect, but no mediating effect on life satisfaction. Finally, a third study comprising 371 secondary students (14.12, SD = 1.78) demonstrates the existence of a serial multiple mediation from gratitude and meaning in life over the relationship between optimism and life satisfaction. Implications are discussed in terms of how prospective variables like dispositional optimism may lead to an increase in subjective well-being (SWB) through different affective and cognitive mechanisms.

## Introduction

Over the last years, there has been increasing interest in the implications of the prospective predictions of human beings (Baumeister et al., 2016). Prospection (mental representation of possible future) is a mental activity that people constantly perform and a fundamental resource to build the present (Gilbert and Wilson, 2007). Consequently, more and more scientific literature has been devoted to the understanding of how prospection allows us to set our objectives and actions and to organize our cognitive and affective processes (Seligman et al., 2013).

Dispositional optimism is deemed one of the most relevant prospective constructs as it refers to the positive expectations we hold for the future and is related to our own motivational processes (Carver and Scheier, 2014). The concept of dispositional optimism differs conceptually and empirically from unrealistic optimism, which stems from the unjustified belief that a personal result will be more favorable than what objective signs indicate (Shepperd et al., 2015).

One of the measures most used to assess dispositional optimism is the Life Orientation Test developed by Scheier and Carver (1985). The model on which the scale was based considered both pessimism and optimism prospective measures. These two measures take into account the general expectations for the life events of a person throughout life. By means of this test, a number of studies linked optimism to different well-being measures based on the hedonic and eudaimonic approaches (Nes and Segerstrom, 2006). For example, optimism is related to hedonic indicators such as experienced positive affect (Carver and Scheier, 1998), but also to eudaimonic measures like psychological well-being (Scheier et al., 2001; Carver et al., 2005).

Nevertheless, despite all the literature relating both constructs, there is still lack of scientific evidence on the mediating mechanisms that explain the complex relationship between the prospective expectations for future and the well-being of the human being (Margolis and Lyubomirsky, 2018).

### Optimism and Life Satisfaction

Optimism behaves as a dispositional trait and presents strong relations with four out of five personality factors: emotional stability, extraversion, agreeableness, and conscientiousness (Sharpe et al., 2011). Optimists often create positive expectations for what is going to happen and anticipate positive outcomes (Scheier and Carver, 1985; Peters et al., 2016). Therefore, optimism is considered an important indicator to promote a subjective well-being (SWB) (Alarcon et al., 2013; Carver and Scheier, 2014). Conversely, pessimists expect negative events when they think of the future. Therefore, pessimism has been often linked to stress and negative feelings such as stress, anxiety, and anger, among other mental health problems (Scheier and Carver, 1992; Carver and Scheier, 1998).

These differences between optimists and pessimists in the way of interpreting expectations for the future have led researchers to refer to the “optimism bias” (Sharot, 2011). In the optimism bias, three cognitive modes explain why optimistic people create positive expectations (Hecht, 2013): (1) selective information processing (i.e., optimistic people direct their attention to positive aspects and tend to ignore the negative ones), (2) locus of control (i.e., optimists tend to believe they own their decisions and trust their own capacities), and (3) attribution styles (i.e., optimists attribute their achievements to internal and stable factors). Different studies have shown that this “optimism bias” is related to increased life satisfaction (Karademas, 2006; Bailey et al., 2007), but the knowledge about the mechanisms activated by optimism, which mediate the relationship between this prospective construct and the subjective perception of life as a whole, is incomplete (Zhang et al., 2014; Margolis and Lyubomirsky, 2018). In a recent review study, Diener et al. (2018a) suggest that optimism predicts SWB because optimism directs people to find meaning in life through different cognitive processes. In this line, this study aims to demonstrate how optimism may provide resources that also increase life satisfaction.

### Optimism, Self-Control, and Grit

Pessimistic and optimistic people differ considerably in their capacity for making plans and setting goals (Carver et al., 2010; Segerstrom et al., 2017). Goal planning and achieving gives meaning to the actions human beings perform in their everyday life (Fowers et al., 2010; Lapierre et al., 2017). From a prospective point of view, thinking about the future implies preparing to act before situations that may appear in order to lead events to expected outcomes (Baumeister et al., 2016). In this sense, one of the aspects that differentiate optimists from pessimists is the situational and general coping styles used to face difficult or stressful situations (Scheier et al., 2001). Previous studies have shown that optimists are more capable of changing stressful circumstances and adapting to them, while pessimists usually use more cognitive avoidance, which generates more stress when facing adverse circumstances (Schou et al., 2005). Additionally, in these negative scenarios, pessimists tend to create negative expectations and to deny reality (Carver and Scheier, 2001).

Besides the differences observed in coping with stress, another important difference between these two groups is the relevance they attribute to goals and values (Segerstrom et al., 2017). Goals not only are generated under stress but also give human beings structure and meaning (Nes and Segerstrom, 2006). Recently, several studies have established self-control and grit as two constructs essential to understanding how people relate themselves to their own goals and progress toward them, promoting their own self-efficacy (Duckworth and Gross, 2014; Hofmann et al., 2014). Self-control is the capacity for resisting immediate temptation, while grit is the ability to persevere in reaching goals in the long term (Tangney et al., 2004; Oriol et al., 2017). One of the most important aspects in studying these constructs is to understand how both are related to goals at higher and lower levels (Eskreis-Winkler et al., 2016). Self-control enhances lower order goals that help individuals achieve short-term goals. Grit is associated with the highest-level and most important goals (Duckworth and Gross, 2014), which implies that people with high grit (long-term) do not always show high self-control (short-term) and vice versa (Duckworth, 2016). Optimistic people create expectations and prospective desires that might act as motivational mechanisms for accomplishing “lower” and “higher” order goals and, consequently, it is especially relevant to analyze their relationship with self-control and grit. Optimism is related to greater commitment to reaching goals as well as greater control over future situations (Geers et al., 2009; Kleiman et al., 2017), but it will be also interesting to observe whether this dispositional variable has a similar effect to that of effort on the achievement of short-term everyday goals and to that of perseverance and interest on accomplishing objectives in the long term. Self-control enables avoiding and facing motivational conflict and improves objective indicators of success, potentiating an increase in perceived well-being (Hofmann et al., 2014), while grit assists in deciding to meet a target in the long term despite the risk of failure and obstacles (Hofmann et al., 2014; Vainio and Daukantaitė, 2016). Thus, we expect that both grit and self-control will have a mediating effect between optimism and life satisfaction.

### Optimism and Positive Affect

One of the most important resources through which optimism seems to contribute to SWB is the positive affect experienced in daily life. According to Diener (2000), people have greater levels of SWB the more the pleasant experiences and the less the painful experiences they have. In this sense, optimistic people experience more positive affect and happiness as they focus more on their success expectations and use positive coping strategies, as opposed to pessimistic people, who concentrate more on anguish and adversity (Schütz and Baumeister, 2017; Segerstrom et al., 2017).

Segerstrom and Sephton (2010) confirmed this assumption by testing the hypothesis that optimism influences positive affect directly in such a way that when optimism increases, so does positive affect. In this line, the bottom-up perspective for SWB emphasizes the importance of experienced positive affect in daily life activities to increase the positive appraisal of our own lives (Kahneman and Deaton, 2010). This perspective is in agreement with “the broaden and build theory” posited by Fredrickson (1998; 2001; 2013), which proposes that positive affect helps individuals “build” enduring personal resources that could increase their SWB (Weinstein and Ryan, 2010; Nelson et al., 2015; Extremera and Rey, 2018). Following these perspectives, optimistic people might experience greater levels of life satisfaction due to positive affect. Thus, we believe that positive affect will have a mediating effect on the relation between optimism and life satisfaction.

### Optimism, Prosociality, and Meaning in Life

Recently, prosociality has been hypothesized as a basic psychological need for the human being (Martela and Ryan, 2016), which would be complementary to the other three needs (relatedness, autonomy, and competence) comprised by the Self-Determination Theory (SDT) (Ryan and Deci, 2000). Therefore, over the last years, increasing attention has been paid to how prospective variables like optimism are related to prosociality (Baumsteiger, 2017). In a recent study conducted by Maki et al. (2016), the authors concluded that prospection promotes prosocial intentions and consequently increases the probability that people help others. Optimists usually have better connections, but they also make an effort to have good relationships (Segerstrom, 2007; Carver and Scheier, 2014). This appears to be because optimism allows us to mobilize positive affective resources that act as drivers and motivational mechanisms for transcending self-interest (Carver and Scheier, 2014). In this sense, one of the most studied emotions that transcends individual interests and that is more relevant to prosociality is gratitude (Haidt, 2003; Stellar et al., 2017). Gratitude refers to acknowledging or being grateful for benefits received from others (Nelson et al., 2016). Different studies point to a strong relation between optimism and gratitude (Emmons and Shelton, 2002; Rey and Extremera, 2014); however, whether optimism and other prosocial variables increase gratitude has not been determined yet. This would be especially relevant to understand if optimism is related to life satisfaction through prosociality, considering that establishing interpersonal relationships builds primary sources of meaning and human strength (Fredrickson et al., 2008; Hicks et al., 2010; Stavrova and Luhmann, 2016) and therefore is fundamental to SWB (Diener et al., 2018b). Concretely, self-transcendence emotions like gratitude are crucial to enjoying experiences and consequently lead to more meaningful views of the world (Lambert et al., 2009; Van Cappellen et al., 2013). Helping and being grateful can make people feel proud and foster a sense of meaning in life (Nakamura, 2013). Thus, optimistic people experience more gratitude, which could give more sense to their lives and, in turn, enhance life satisfaction. From the eudaimonic perspective of well-being, for people to experience long-lasting happiness, they need to live a life full of meaning (Ryan and Deci, 2008). In this line, different studies have shown the importance of meaning in life to the increase in life satisfaction (Steger et al., 2008; Steger, 2018).

## Present Studies

To deepen the knowledge about the mechanisms mediating the relation between optimism and life satisfaction, we conducted three studies. Study 1 assesses the relationship between optimism and life satisfaction through self-control and grit. The two later constructs are key to understanding how human beings achieve their goals and succeed in their daily lives (Duckworth and Gross, 2014). It should be noted that pursuing and attaining individually important goals is a central aspect for SWB (Diener, 1984). Therefore, this first study seeks to observe how dispositional optimism acts as a potential driver of these two constructs in their relation to successful goal pursuit, promoting an increase in life satisfaction.

H1 Dispositional optimism will be positively associated with life satisfaction through self-control.

H2 Dispositional optimism also will be positively associated with life satisfaction through grit.

The purpose of study 2 was to analyze how dispositional optimism is related to life satisfaction considering positive affect a mediator. As indicated in the broaden and build theory proposed by Fredrickson (2013), positive affect contributes to motivational processes and personal resources vital to people’s well-being. Specifically, we seek to prove that optimism promotes the experience of positive affect and thereby increases the more cognitive component of SWB.

H3 Dispositional optimism will be positively associated with life satisfaction through positive affect.

Finally, study 3 will attempt to demonstrate how optimism is linked to life satisfaction through two extremely relevant variables from a eudaimonic well-being perspective, namely, gratitude and life meaning (Ryan et al., 2013). Particularly, a serial multiple mediation is expected when taking gratitude as a predictor of life meaning.

H4 Dispositional optimism will be positively associated with life satisfaction through a serial multiple mediation model composed by gratitude and meaning in life.

## Study 1

### Methods

#### Participants

Since data collection was conducted at one specific time, the nature of this study is cross-sectional. Two hundred seventy-five secondary students from Lima Metropolitana, Peru, participated in this study (132 women, 50.8%, and 128 men, 49.2%). The mean age of these students was 14.82 (SD = 1.07 years of age). The sample is part of the instrument validation pilot plan from the program “Escuela Amiga,” which was developed by the Education Ministry of Peru (MINEDU). The objective of this program was to improve the socioemotional skills of primary and secondary students in 350 public schools from Lima Metropolitana (Oriol et al., 2017).

The Education Quality Directorate of Peru’s Education Ministry was the unit in charge of supervising the ethical dimension and implementation of the study with the technical support of the World Bank. To apply the pilot survey, informed consent forms were created for parents and guardians. These forms explained the objectives of the study and were based on the ethical guidelines of the Declaration of Helsinki. Before applying the questionnaires, the objective of the study was also described to the students, who signed an informed consent form.

Regarding the ethical considerations for applying the survey, the students’ parents or guardians were requested to sign an informed consent. Likewise, protocols were designed in conjunction with school psychologists to offer emotional support for those students who might feel distressed when answering the questionnaires.

#### Measures

The instruments employed correspond to the instruments used by the “Escuela Amiga” project. Prior to the survey, a qualitative validation was conducted, and experts were consulted to assess cultural adequacy in the scales. The qualitative validation consisted of three focus groups with secondary students, while experts were asked to classify the scale items as essential, adequate, or inadequate. Two complementary analyses were made from the information gathered. First, all instruments were analyzed by subdimension to evaluate whether the information obtained from the set of instruments measures the corresponding subdimensions comprehensively. The content validity coefficient (CVC) was used to this end. Second, instruments were assessed once more separately to measure the degree of agreement between the evaluations of the external observers. Kappa coefficient was used at this stage. Indexes were satisfactory for both coefficients: 0.8 and 0.73 for Kappa and CVC, respectively (Hernández-Nieto, 2002).

The following scales were used in this article:

##### Optimism

The optimism scale is part of the Middle Years Development Instrument (MDI) and presents suitable psychometric indicators (Schonert-Reichl et al., 2013). It is composed of three items (e.g., “In the future, I think I’ll have more good times than bad times” and “I start most days thinking I will have a good day”). These items were assessed through a five-point Likert scale, from 0 (“Totally disagree”) to 4 (“Totally agree”) (*M* = 2.64, SD = 0.74; α = 0.71). Following the recommendations proposed by Byrne (2013), we did not conduct a confirmatory factor analysis because when instruments have only three items, the model is saturated (the number of observed variables is the same as the number of parameters to be calculated) and therefore values equal to 1 are obtained in indexes such as Comparative Fit Index (CFI) and Tucker–Lewis Index (TLI).

##### Life satisfaction

It is a scale developed by Gadermann et al. (2010), which is composed of the following five items: “In most ways, my life is close to the way I would want it to be”; “The things in my life are excellent”; “I am happy with my life”; “So far, I have got the important things I want in life”; and “If I could live my life over, I would have it the same way.” This scale is based on the cognitive processes children and adolescents undergo in terms of the satisfaction they feel with life. The scale is complementary to the single-item scale proposed by Diener et al. (1999). All these variables are measured through a five-point Likert scale ranging from 0 to 4 (0 = “Totally disagree” and 4 = “Totally agree”) (*M* = 2.38, SD = 0.84; α = 0.78). A first confirmatory factor analysis was conducted, and some adequate adjustment indexes were obtained; however, root mean square error of approximation (RMSEA) was too high (TLI = 0.91, CFI = 0.95, RMSEA = 0.11). Thus, error covariances were computed between item 4 “So far, I have got the important things I want in life” and item 5 “If I could live my life all over again, I would change almost nothing” in order to obtain better adjustment indexes (TLI = 0.91, CFI = 0.95, RMSEA = 0.08).

##### Grit

Based on the Grit Scale of Duckworth and Quinn (2009), this scale is part of the assessment instrument employed in the program “Escuela Amiga” led by the Education Ministry of Peru. The instrument above was called CUBE or *School Well-Being Single Questionnaire* and was composed of eight items divided into two dimensions: perseverance (e.g., “I always finish what I start” and “I am dedicated and careful”) and effort (e.g., “I often set a goal, but I look for a different one later” and “I find it difficult to keep interest in things that need many months to be finished”). The scale is assessed through a five-point Likert scale whose answer range is similar to that of the Life Satisfaction Scale (*M* = 2.17, SD = 0.54; α = 0.70). Nevertheless, after removing the item “Difficulties when something takes time or doesn’t work do not discourage me,” Cronbach’s alpha improves to 0.75. Likewise, to reach adequate adjustment indexes, the item “I have become enthusiastic about ideas or projects, but I lose interest soon after” is deleted. By this action, the reported adjustment indexes were TLI = 0.90, CFI = 0.95, RMSEA = 0.07.

##### Self-control

It is a scale based on the self-regulation inventory created by Moilanen (2007). The scale comprises four items that assess the regulation dimension in the short term. These items are related to emotional and attentional regulation (e.g., “I’m able to calm down when I’m excited or upset” and “If something doesn’t go as planned, I do something different to achieve my objective”). This scale is assessed on a five-point Likert scale, whose answer range is similar to the scales described above (*M* = 2.60, SD = 0.68; α = 0.74). A first confirmatory factor analysis was conducted and, as a result, some indexes showed inadequate values (TLI = 1.00, CFI = 1.00, RMSEA = 0.01). Therefore, the covariance between the errors of both items and an acceptable adjustment was achieved (TLI = 0.97, CFI = 0.97, RMSEA = 0.01).

#### Methodology

The variables in this study have a normal distribution because the *z*-test using skewness and kurtosis presents absolute values below 3.29 (Kim, 2013). Regarding the means of the measures, variables score higher than the midpoint of the scale (>2) (Table 1). As for correlations between variables, all are positive and significant (*p* < 0.05), but life satisfaction is associated with other variables only moderately.

**TABLE 1:** Descriptive and correlational statistics between the variables of study 1.

	**Min**	**Max**	**M**	**SD**	**1**	**2**	**3**	**4**
1. Optimism	0	4	2.64	0.74	–			
2. Life Satisfaction	0	4	2.38	0.84	0.45**	–		
3. Grit	0	4	2.18	0.54	0.28**	0.44**	–	
4. Self-Control	0	4	2.51	0.69	0.35**	0.48**	0.48**	–

** The correlation is significant at 0.01 (bilateral).

The results of study 1 are divided into two parts. The first part shows the results of the explanatory model for life satisfaction in relation to optimism and the mediating variable grit. In the second part, the explanatory model takes self-control as a mediating variable. Structural equation modeling (SEM) was conducted using AMOS v22. This technique offers the advantage of testing complex multivariate models while controlling for the measurement error of the variables studied. Bootstrapping was used in this analysis.

The sample surveyed complies with the recommendations of Bentler and Chou (1987) for analyzing structural equations. The authors suggest that the ratio between sample size and free parameters should be close to 5:1. Values below 5 are considered acceptable for the χ^2^/g.l coefficient. In the case of RMSEA, 0.08 indicates an acceptable fit, and values below 0.05 indicate a good model fit (Hu and Bentler, 1999). Additionally, values above 0.90 indicate an acceptable fit for CFI and TLI.

### Results

Table 1 presents the descriptive results of the variables analyzed. Correlations are positive and significant between the variables under study.

#### Grit and Self-Control as Mediator Variable Between Optimism and Life Satisfaction

First, an SEM model was calculated considering both variables (grit and self-control) as mediator variables. After computing the measurement model, the covariance between grit and self-control was extremely high (0.88), and consequently, adjustment indexes were inadequate. Due to the lack of adjustment, we decided to try two separate models, which had both grit and self-control as only mediators of each model.

#### Grit as Mediator Variable Between Optimism and Life Satisfaction

**FIGURE 1:**
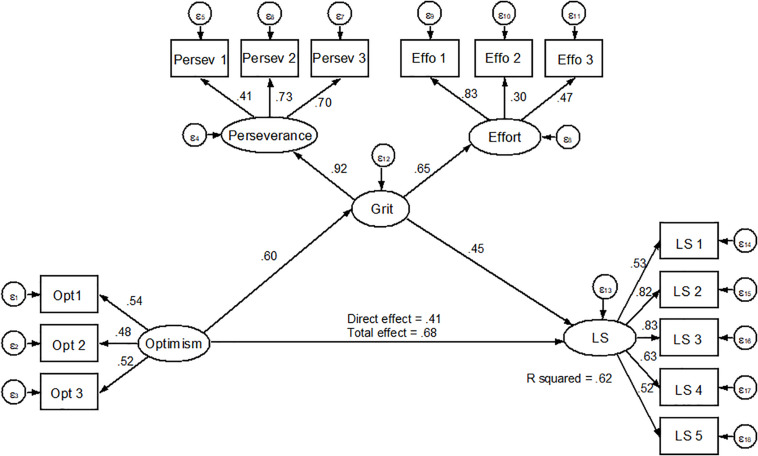
Structural equation model for grit as mediator variable.

Prior to the calculation of the first SEM, the measurement model was computed. For adjustments in the general measurement model, the assessed indexes are χ^2^ = 186.982, χ^2^/g.l. = 8.44; TLI = 0.88, CFI = 0.90, RMSEA = 0.049. Likewise, the analysis of the factor loadings of the items in the measurement model shows that two items from the grit dimension have loads below 0.3. These items were eliminated from the latent variable, and adjustment indexes improved in this new measurement model (χ^2^ = 135.489, χ^2^/g.l. = 10.44; TLI = 0.91, CFI = 0.93, RMSEA = 0.058) (see Figure 1).

The structural equation model for optimism, which predicts life satisfaction mediated by grit, while controlling for age and gender, shows satisfactory adjustment indexes (χ^2^ = 163.480, χ^2^/g.l. = 8.23; TLI = 0.91, CFI = 0.93, RMSEA = 0.05). The model explains 62% of life satisfaction’s variance. In this model, error terms were not correlated to obtain better adjustment indexes.

As expected, according to the reported standardized estimators, the effect of optimism over grit was β = 0.60, *p* < 0.05. On the other hand, regarding satisfaction with life, grit predicts positively and significantly life satisfaction (β = 0.45, *p* < 0.05), while the relationship between optimism and life satisfaction, which is mediated by grit, has an indirect effect that is positive and significant (β = 0.27, *p* < 0.05).

#### Self-Control as Mediator Variable Between Optimism and Life Satisfaction

In this model, the mediating variable grit was replaced by self-control. As in the previous model, the measurement model was calculated, yielding acceptable adjustment indexes (χ^2^ = 109.986, χ^2^/g.l. = 9.82; TLI = 0.91, CFI = 0.93, RMSEA = 0.05).

**FIGURE 2:**
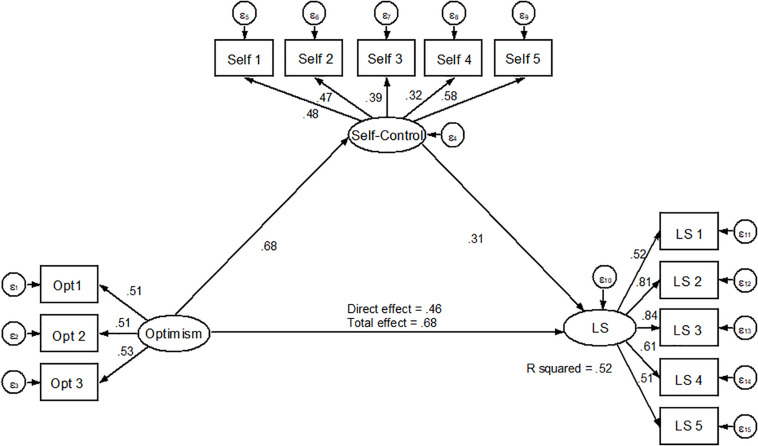
Structural equation model for self-control as mediator variable.

Regarding the structural equation model in Figure 2, it is seen that the effect size of optimism over self-control predicts positively (β = 0.68, *p* < 0.05), while self-control predicts life satisfaction positively as well (β = 0.31, *p* < 0.05). As for the relationship between optimism and satisfaction mediated by self-control, the indirect effect reported is β = 0.28, *p* = 0.09.

## Study 2

### Methods

#### Participants

A cross-sectional study was conducted with university students. Specifically, 1,365 university students from the Peruvian government “Beca 18” program took part in this study, out of which 57.7% were men (*n* = 784), with a mean age of 21.5 years (SD = 2.35). The sample assessed is part of the psychometric validation process for the socioemotional well-being questionnaire designed and implemented by the National Scholarship Program of the Education Ministry of Peru (PRONABEC).

This study was designed and conducted by the Recipient’s Well-Being Office of PRONABEC (Education Ministry of Education). Experts from different fields working in the Well-Being Offices of the Education Ministry evaluated the ethical suitability following the Helsinki Declaration guidelines. Prior to filling in the form, students were asked to consent the questionnaire application. Students who agreed to participate were informed that the survey was confidential and that they did not need to answer all the questions, but only the ones they were willing to. The questionnaire was uploaded to a virtual platform, and the link was sent to a randomized sample of students from the program.

#### Measures

##### Optimism

As in study 1, the optimism scale was used from the MDI (Schonert-Reichl et al., 2013), which is composed of three items assessed in a five-point Likert scale (*M* = 3.05, SD = 0.64; α = 0.78).

##### Positive affect

It is a scale based on the 10-item Positive and Negative Affect Scale for Children (PANAS-C10) developed by Damasio et al. (2013). This scale assesses five items related to positive affect (happy, cheerful, fun, content, and joyful) and five items associated with negative affect (humiliated, bothered, irritated, hurt, and sad). PANAS evaluates to what extent participants have experienced any of the emotions above during the present week. The scale is measured in a five-point Likert scale that ranges from 0 = “Very little or nothing” to 4 = “Extremely.” This paper used the subdimension positive affect (*M* = 2.67, SD = 0.71; α = 0.88). The reported adjustment indexes were TLI = 0.98, CFI = 0.98, and RMSEA = 0.05.

##### Life satisfaction

The Brief Multidimensional Students’ Life Satisfaction Scale (BMSLSS) was used. This scale has five items, each of which assesses a different domain of life satisfaction (Seligson et al., 2003). These items measure to what extent students feel “dissatisfied” or “satisfied” with the following aspects of their lives: (1) family life, (2) friendships, (3) life at school/institute, (4) themselves, and (5) the place where they live. Originally, the scale assessed satisfaction with school, but it was adapted because the population surveyed is in higher education. This scale employs a seven-point Likert scale where 0 = “Very dissatisfied” and 7 = “Very satisfied” (*M* = 4.90, SD = 1.01; α = 0.81). The adjustment indexes reported for this scale were TLI = 0.95, CFI = 0.97, and RMSEA = 0.08.

#### Methodology

The moderation analyses were conducted using AMOS 22, and an SPSS plug-in was employed for the SEM. This technique offers the advantage of testing complex multivariate models, while controlling for the error measures of the variables. All SEM analyses consider the full structural model, including its corresponding items. Bootstrapping was used for the moderation analysis. According to the recommendations of Bentler and Chou (1987), an RMSEA value of 0.08 indicates an acceptable fit, and values below 0.05 indicate a good model fit. Additionally, values above 0.90 indicate an acceptable fit for CFI and TLI (Hu and Bentler, 1999). The error terms of the calculated models were not correlated.

### Results

The variables included in the study have a normal distribution. Additionally, there are relatively small and moderate correlations between the variables, which indicate little multicollinearity. In Table 2, the correlations between the well-being indicators and optimism (*r* = 0.48) and well-being and positive emotions (*r* = 0.49) are moderate.

**TABLE 2:** Descriptive and correlational statistics for the variables of study 2.

**Variable**	**Min**	**Max**	**M**	**SD**	**1**	**2**	**3**
1. Optimism	0	4	3.04	0.64	(0.78)^a^		
2. BMSLSS	0	6	4.9	1.02	0.48**	(0.90)^a^	
3. Positive affect	0	4	2.67	0.71	0.48**	0.49**	(0.88)^a^

^*a*^Cronbach’s alpha. ** The correlation is significant at 0.01 (bilateral).

The second part of results shows the mediating effect of positive emotions over the relationship between optimism and BMSLSS.

#### Structural Equation Model for Life Satisfaction

BMSLSS, Brief Multidimensional Students’ Life Satisfaction Scale.

To verify that the model and the variables analyzed are adequate, the measurement model was calculated before the SEM. The measurement model had an adequate fit (χ^2^ = 360.85, χ^2^/g.l. = 4.88; TLI = 0.98, CFI = 0.98, standardized root mean residual (SRMR) = 0.05).

Regarding the fit of the structural equation model, the adjustment indices also present adequate values, with χ^2^ = 360.85, χ^2^/g.l. = 4.87; TLI = 0.98, CFI = 0.98, and SRMR = 0.053. In this model, optimism predicts positive emotions (β = 0.57, *p* < 0.05), as well as positive emotions do with life satisfaction (β = 0.32, *p* < 0.05).

**FIGURE 3:**
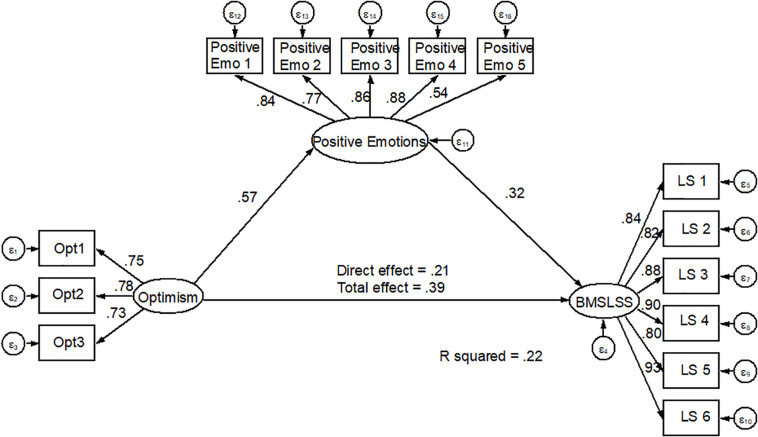
Mediated structural equation model for optimism over BMSLSS through positive affect.

Moreover, the mediating effect of positive emotions on the relationship between optimism and life satisfaction has a significant indirect effect (β = 0.18, *p* < 0.05), and the model explains 22% of variance over BMSLSS (see Figure 3).

## Study 3

### Methods

#### Participants

Study 3 is part of another cross-sectional study carried out by the Rural Training Centre Switching (RTCS) programs overseen by the Education Ministry of Peru. RTCSs are public education institutions located in rural areas where populations of different sizes live and whose economic activities are mainly agricultural or livestock activities, as well as forestry and small production of goods and services. The creation of these centers has been led by local householders and actors, who develop joint work with local and regional education institutions in order to provide comprehensive education to adolescents and young people in the area. Three hundred seventy-one students participated in this study: 48.8% of women (*n* = 181) with a mean age of 14.1 years (SD = 1.78) belonging to RTCSs. The sample surveyed corresponds to the baseline of socioemotional skills for the project, which was led by MINEDU. Specifically, the Rural Services Directorate of the Education Ministry of Peru was the institution responsible for conducting this study. Data analyzed in this article correspond to the psychometric assessment of the emotional skills and well-being questionnaire for students from MINEDU’s rural service. Workers from such a Ministry designed the methodology and ethical considerations of the study considering the Declaration of Helsinki. Due to the characteristics of the education service, whose main component is boarding schools, MINEDU also asked school principals for their informed consent to apply the questionnaire, in addition to the students’ assent. Students were also told that participation in the study was voluntary and that the answers provided would be anonymous and confidential.

#### Measures

##### Optimism

As in studies 1 and 2, the optimism scale was adapted from the MDI Inventory. This scale is composed of three items assessed in a five-point Likert scale (*M* = 3.04, SD = 0.68; α = 0.71).

##### Gratitude

This scale was adapted from the Gratitude Questionnaire for youth (Froh et al., 2011). Items such as “I like to return favors,” “I like to thank people,” “I appreciate when others help me,” and “Every time I can, I return the favors received” are assessed through a five-point Likert scale, in which 0 = “Totally disagree” and 4 = “Totally agree” (*M* = 3.49, SD = 0.48; α = 0.74). For this scale, adjustment indexes were TLI = 0.91, CFI = 0.97, and RMSEA = 0.09.

##### Life satisfaction

As in study 1, this scale was developed by Gadermann et al. (2010) with five items. It includes the single item proposed by Diener et al. (1999). All these variables are measured through a five-point Likert scale that ranges from 0 (“Totally disagree”) to 4 (“Totally agree”) (*M* = 2.73, SD = 0.70; α = 0.70). The adjustment indexes reported for this scale were TLI = 0.90, CFI = 0.95, and RMSEA = 0.09.

##### Meaning in life

Based on Steger et al. (2006), this scale has 10 items in two dimensions: presence (e.g., “I am always looking to find my life’s purpose) and search (e.g., “I am looking for something that makes my life feel meaningful”). The scale employs a five-point Likert scale that ranges from 0 (“Totally disagree”) to 4 (“Totally agree”) (*M* = 3.06, SD = 0.54; α = 0.76). This scale reports the following adjustment indexes: TLI = 0.90, CFI = 0.92, RMSEA = 0.08.

#### Methodology

In the same line of studies 1 and 2, the third study conducted the moderation analyses using AMOS 22. Based on the adequate adjustment indexes proposed by Hu and Bentler (1999), RMSEA values of 0.08 indicate an acceptable fit, and values below 0.05 indicate a good model fit. Additionally, values above 0.90 indicate an acceptable adjustment for CFI and TLI.

### Results

As in the previous studies, all SEM analyses consider the full structural model, including its corresponding items. Additionally, bootstrapping was used for moderation analysis.

Table 1 shows that all scores are above the midpoint of the scale, with gratitude having the highest score (*M* = 3.49, SD = 0.48). In terms of correlations between variables, the relationship between meaning of life and optimism (*r* = 0.51) and between meaning of life and gratitude (*r* = 0.57) is the strongest, while correlations over the relation between life satisfaction and optimism (*r* = 0.29) and between gratitude (*r* = 0.28) and meaning of life (*r* = 0.33) are moderate (see Table 3).

**TABLE 3:** Descriptive and correlational statistics between the variables of study 3.

**Variable**	**Min**	**Max**	**M**	**SD**	**1**	**2**	**3**	**4**
1. Life satisfaction	0	4	2.73	0.70	(0.67)^a^			
2. Optimism	0	4	3.04	0.68	0.29**	(0.51)^a^		
3. Meaning of life	0	4	3.06	0.54	0.33**	0.51**	(0.76)^a^	
4. Gratitude	0	4	3.49	0.48	0.28**	0.42**	0.57**	(0.71)^a^

^*a*^Cronbachs alpha. ** The correlation is significant at 0.01 (bilateral).

As in studies 1 and 2, mediation analyses were used to test the hypotheses through SEM. First, the measurement model was analyzed, presenting adequate adjustment indexes (χ^2^ = 361.09, χ^2^/g.l. = 9.32; TLI = 0.92, CFI = 0.92, SRMR = 0.05).

Regarding the structural equation model with sequential mediation, adjustment indexes are adequate (χ^2^ = 360.85, χ^2^/g.l. = 10.10; TLI = 0.91, CFI = 0.91, SRMR = 0.05). In this model, there is a standardized effect of optimism over gratitude (β = 0.81, *p* < 0.05), gratitude has a standardized effect over meaning of life (β = 0.80, *p* < 0.05), and meaning of life predicts significantly life satisfaction (β = 0.27, *p* < 0.05).

**FIGURE 4:**
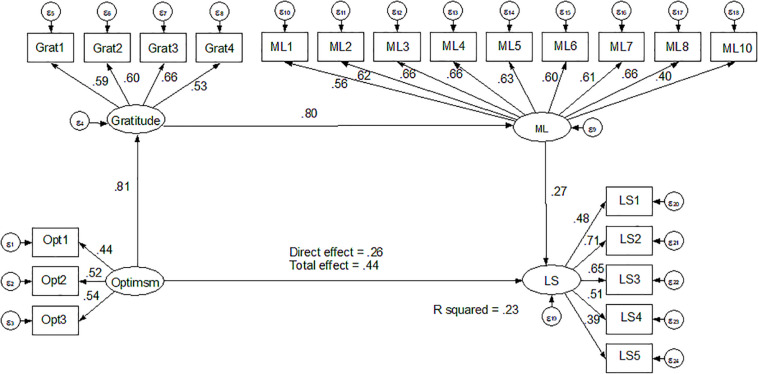
Serial multiple mediation structural model of optimism over LS.

Likewise, the mediating effect of meaning of life on the relation between gratitude and life satisfaction was also significant (β = 0.21, *p* < 0.05). The model explains 23% of variance in life satisfaction (see Figure 4).

## Discussion

This study aimed to demonstrate through three studies how a prospective variable like optimism relates with life satisfaction through the activation of successful goal pursuit (self-control and grit), positive affect, gratitude, and meaning in life. According to the first hypothesis, grit mediated the relation between optimism and life satisfaction, but no significant indirect effect was found in the case of self-control. These results have different implications. Optimism is related to both self-control and grit; therefore, as suggested by previous studies, it promotes commitment to short-term goals (Carver and Scheier, 2001; Nes and Segerstrom, 2006), but also the tenacity to achieve goals in the long term. Precisely, according to our results, tenacity and perseverance with long-term objectives exhibit a mediating effect on life satisfaction. These data indicate that, besides helping to deal with stressful situations as suggested by the literature (e.g., Scheier et al., 2001), these variables are relevant for people to achieve their goals and high-order objectives, which enhances their well-being perception. In this line, the SDT developed by Ryan and Deci (2000) underscores the psychological need of human beings to reach the goals they have proposed themselves. Positive expectations are an essential element to activate motivational processes, but results point out that perseverance to accomplish objectives in the long term is necessary for well-being. Objectives promote structure, sense, and identity, while grit implies the ability to maintain effort and interest to meet these objectives in spite of failure and adversity (Duckworth and Quinn, 2009).

Following the second hypothesis, positive affect mediates the relationship between optimism and life satisfaction. Thus, results support the idea of a bottom-up effect, which would imply that positive affect influences the global cognitive appraisal people make of their own well-being. The SWB model proposed by Diener (2000) emphasizes the relevance of both the cognitive and affective components for SWB. Nevertheless, some authors suggest that a bottom-up effect helps understand the construction of a more global appraisal when asking about life satisfaction (Diener et al., 2018a). According to Fredrickson (2003), the tendency to experience positive emotions promotes the accumulation of personal resources and thus contributes to the construction of SWB as well. Other authors such as Kahneman and Deaton (2010) also highlighted the influence of experienced positive affect on the cognitive appraisal of people. In this vein, our results confirm the predisposition of optimism toward the experience of positive affect. However, it must be noted the relevance that the constant accumulation of positive affect may have to do with a more global notion of satisfaction with different aspects of life.

For the third study, we proposed a serial multiple mediation model, which examined gratitude and meaning in life as mediators in the relationship between optimism and life satisfaction. Results show a strong relationship between optimism and gratitude. This is especially relevant considering that a recent literature review indicates that thinking about the future could promote prosociality (Maki et al., 2016; Baumsteiger, 2017). In addition, variables related to prosociality like gratitude, which promotes self-transcendence, seem to be key to human well-being (Schwartz and Sortheix, 2018). According to the SDT developed by Ryan and Deci (2000), debate has sparked recently about the need of demonstrating that prosociality is a basic need of the human being (Martela and Riekki, 2018). Our results do not answer this question but clearly point out that gratitude plays an important role in the promotion of life meaning and that both variables enhance the global perception of well-being. These results confirm the findings of a recent study by Martela and Ryan (2016), in which prosociality promoted meaningfulness and, in turn, SWB. Transcendence emotions like gratitude contribute to maintaining interpersonal relationships in the long term (Algoe, 2012) and therefore help human beings to build personal, physical, and mental resources in the long term (Fredrickson, 2003).

In sum, results show that: (1) Optimism is strongly related to life satisfaction in two studies with different samples. Thus, this supports the idea that this prospective trait is key to increasing SWB, as pointed out by previous studies (Ho et al., 2010; Duy and Yildiz, 2017). (2) Optimism acts as a driving mechanism for both self-control and grit. In turn, self-control mediates the relationship between optimism and life satisfaction. Optimism and grit contribute to explain 62% of the variance of life satisfaction, while optimism, and self-control explain 52%. (3) Results from study 2 indicate that optimism is related significantly and positively to positive affect, which exhibits a mediating effect between optimism and life satisfaction. Specifically, optimism and positive affect explain 22% of the variance of life satisfaction. (4) Optimism also shows a strong relation with gratitude, and gratitude and meaning in life have a mediating effect on the relation between optimism and life satisfaction. Specifically, optimism, gratitude, and life meaning explain 23% of the variance of life satisfaction.

## Limitations

As a limitation of this study, we should mention that the three studies are cross-sectional, and therefore, longitudinal research is needed to observe how expectations for the future affect these cognitive and affective mechanisms across time. The limitation of using transversal data is relevant to the mediation analysis, according to Maxwell and Cole (2007).

Regarding participants’ age, two of the studies were conducted with adolescents and the third with a sample composed of university students. In this sense, delving into the relationship between the two constructs by considering the different steps of development is necessary. Finally, the study was comprised of possible mediators, but other mediating effects from other variables should be included in the relationship between both constructs.

## Conclusion

The relationship between dispositional optimism and different well-being measures has been studied for several years. However, optimism has been recently integrated as one of the relevant constructs in the work line of prospective studies (MacLeod, 2017), which has raised questions about how these predictive variables can contribute to the increase of the SWB of human beings. Results from the three studies indicate a strong relationship between optimism and life satisfaction, as well as with all the mediators comprised by this study. Thus, dispositional optimism acts as a driver or motivational mechanism fundamental to mobilize cognitive and affective resources associated with both hedonic and eudaimonic well-being. In connection, there are differences in the understanding of how dispositional optimism relates to life satisfaction. First, grit helps humans set long-term goals and persevere until their hedonic and eudaimonic wel achievement, aspect that is highly relevant to SWB. Second, variables such as gratitude and meaning in life enable people to relate to each other in a positive way, thereby promoting personal growth.

These results open the doors for more research on how prospection is related to SWB. Furthermore, this stresses the importance of launching programs and actions aimed at developing dispositional optimism from early ages to build the cognitive and affective resources necessary for the promotion of well-being.

## Data Availability Statement

The datasets generated for this study are available on request to the Education Ministry of Peru.

## Ethics Statement

This study was supervised by the Review Board of Education Quality Directorate of Peru’s Education Ministry, following the ethical principles for research involving human participants contemplated in the Declaration of Helsinki.

## Author Contributions

XO: data interpretation, introduction, discussion, and article revision. RM: data collection, procedure, and analyses. CB: literature review and corrections. EB: data collection, article revision, and bibliography.

## Conflict of Interest

The authors declare that the research was conducted in the absence of any commercial or financial relationships that could be construed as a potential conflict of interest.
